# Is employment-focused case management effective for patients with substance use disorders? Results from a controlled multi-site trial in Germany covering a 2-years-period after inpatient rehabilitation

**DOI:** 10.1186/s12888-016-0990-7

**Published:** 2016-08-05

**Authors:** Susanne Saal, Lukas Forschner, Dietmar Kemmann, Jacqueline Zlatosch, Thomas W. Kallert

**Affiliations:** 1 Institute of Health and Nursing Sciences, Medical Faculty, Martin-Luther-University of Halle-Wittenberg, Magdeburger Straße 8, 06097 Halle, Germany; 2 SRH Medinet GmbH Fachklinik Alte Ölmühle, Berliner Chaussee 66, 39114 Magdeburg, Germany; 3 Diakonie-Krankenhaus Harz GmbH, Brockenstraße 1, 38875 Elbingerode, Germany; 4 AHG Klinik Römhild, Am Großen Gleichberg 2, 98630 Römhild, Germany; 5 HELIOS Park-Klinikum Leipzig, Zentrum für Seelische Gesundheit/Soteria Klinik Leipzig, Morawitzstr. 4, 04289 Leipzig, Germany

**Keywords:** Case management, Return to work, Substance use disorders, Controlled trial, Germany

## Abstract

**Background:**

Substance use disorders are associated with unemployment. An employment-focused case management (CMRE) has been conceptualised as a specific intervention to help substance use disorder patients return to competitive employment immediately after inpatient rehabilitation. This study investigated the effect of the intervention on return to work of persons with substance use disorders.

**Method:**

The study was conducted in four German inpatient rehabilitation departments, and included unemployed patients (aged between 18 and 63 years) with a main clinical diagnosis of ICD-10 F10-19 disorders. Six weeks before discharge, patients were randomly allocated to CMRE or standard care (SC) using a quasi-randomised approach. The primary outcome measure was integration into competitive employment 24 months after discharge from rehabilitation. Secondary outcome domains were abstinence, duration of employment, proportion of publicly funded employment, satisfaction with life, precarious housing situation and precarious financial situation, and use of follow-up services. Outcome measures were assessed 6 weeks and 1–2 days prior to discharge, and 12 and 24 months after discharge from rehabilitation.

**Results:**

One hundred sixty patients were allocated into the CMRE group and 160 patients into the control group. 267 resp. 179 participants could be included in the analyses performed for the 12-, and the 24-months follow-up assessments. At the study endpoint the rate of integration into the primary labour market was 35.6 % in the CMRE group and 41.2 % in the control group, respectively (Relative Risk 0.92, 95 % CI, 0.47; 1.79). There was a significantly higher proportion in the CMRE group, however, which immediately after discharge linked with services of the Federal Employment Agency or Job Centres. There were no statistically significant differences in other outcomes between the groups.

**Conclusions:**

Compared to SC, the additional specific CMRE intervention did not result in superior effects on return to work rates, abstinence, satisfaction with life, and housing and precarious financial situation. But CMRE was more effective on linking substance use disorder patients with services of the Federal Employment Agency or Job Centres. Reasons for the finding that such close linking does not have an impact on return to work rates are discussed in detail.

**Trial registration:**

Identifier: DRKS00003574; March 12, 2012. The trial was retrospectively registered.

**Electronic supplementary material:**

The online version of this article (doi:10.1186/s12888-016-0990-7) contains supplementary material, which is available to authorized users.

## Background

Problematic substance use is associated with unemployment, since substance use disorders may elicit absence from work and unemployment but, in reverse, unemployment may lead to substance use disorders [1]. In Germany, more than one third of patients with alcohol abuse disorders and more than two-thirds of patients with drug abuse disorders treated in inpatient substance use rehabilitation departments are unemployed [2, 3]. Furthermore, unemployment increases the risk of relapse after alcohol and drug addiction treatment [1]. Therefore, one of the major purposes of rehabilitation in this field is to re-integrate patients into competitive employment immediately after inpatient rehabilitation. In order to realise this aim, which is important also from the population-based economic and public mental health standpoint of the financial carriers of these measures, standard care of substance use treatment in inpatient rehabilitation departments in Germany offers employment-focused counselling, assessment and training schemes. However, the impact of these efforts on return-to-work rates seems to be limited. Out of all unemployed persons with substance use disorders in inpatient rehabilitation only 5.2 % return to work immediately after treatment [4].

Although the risk of relapse is largest in the first 2 months after discharge from inpatient substance use rehabilitation [3], a close linking between inpatient substance rehabilitation and post-treatment employment measures has still not been established in Germany. Case management is a frequently used method to integrate health-related services across such interfaces [5].

Several trials conducted in the context of substance use rehabilitation have examined effects of case management on outcome domains like abstinence, linkage with and compliance with outpatient long-term treatment measures and auxiliary services. A most recent meta-analysis on this issue [6] analysed data from 21 randomised trials, and found moderate improvements in linkage with and utilization of substance abuse treatment and important auxiliary services, but only weak effects on social inclusion, substance consumption and risk behaviour.

Prior to the beginning of our own trial, a systematic literature search revealed a lack of trials that examined the effects of case management for persons with substance use disorders on employment-focused outcomes. We found two randomised trials assessing such outcome domains [7, 8], searched the reference list of the above-mentioned meta-analysis [6], and thus could identify one additional trial [9]. Case management was examined for persons attending a polysubstance rehabilitation program (*n* = 632) [7], in a pilot study of a Methadone Maintenance program (MMT) (*n* = 23) [8], and in a probation program for drug-involved women offenders (*n* = 183) [9]. None of the studies demonstrated significant overall improvements for the employment outcome measures used (i.e., Addiction Severity Index, employment domains, worked at least 30 days and net income). However, in a subgroup-analysis among the subjects expressing extreme interest in employment issues (*n* = 193) one study found significant differences between the case management and the non-case-management (NCM) groups within a 3-month follow-up period: case-management (CM) clients (*n* = 100) worked more days (15.6 days vs. 12.1 days) than the comparable group of NCM clients (*n* = 93). Furthermore, CM clients reported fewer days with employment problems, feeling “less troubled” about their employment status and seeing “less need” for employment counselling [7]. In summary, the findings of this small number of trials demonstrated the need to further explore the effects of case management on employment outcomes.

Against this background, our trial used a parallel controlled study design, and - in comparison to care as usual - aimed to assess the effects of employment-focused case management provided during and after inpatient substance use rehabilitation, focusing on return to work in a 24-months follow-up period. Encouraged by the results of a pilot study in which the intervention was modelled and pilot-tested without a parallel control group [10], and which showed positive effects of the intervention on employment status and abstinence, we hypothesised that a case management approach focused on employment issues might improve the return to work of persons with substance use disorders. Further, we hypothesised that this intervention might diminish the risk of drug use relapse after discharge from rehabilitation.

## Methods

### Study period, sites, and inclusion criteria

The trial was conducted from September 2011 to September 2014. Participants were recruited consecutively in four inpatient rehabilitation departments in Central Germany (SRH Medinet GmbH Fachklinik Alte Ölmühle Magdeburg, HELIOS Park-Klinikum Zentrum für Seelische Gesundheit/Soteria Klinik Leipzig, AHG Klinik Römhild, and Diakonie-Krankenhaus Harz GmbH Elbingerode) between October 2011 and June 2012. Two inpatient rehabilitation departments were located in rural areas with wide catchment areas (Römhild, Elbingerode); the other two were urban clinics with small catchment areas (Magdeburg, Leipzig). All rehabilitation departments focused on inpatient rehabilitation of patients with substance use disorders aged over 17 years. Due to evidence-based recommendations of the public finance provider, the therapeutic orientation of all the sites is more or less standardized, and comprises group-psychotherapy, one-to-one psychotherapeutic sessions, psycho-education concerning several addiction-related topics, occupational and vocational in-house training, physical therapy, social work, training of recreational activities, and involvement of relatives. In addition, two clinics offer a service called adaption treatment within a 12-week period right after inpatient rehabilitation: patients with a persisting need of vocational and social integration support and without a domicile are given the opportunity of assisted living and practical vocational training in companies on the first labour market and are supervised by trained social workers.

The inclusion criteria were: age between 18 and 63 years, main ICD-10 clinical diagnosis of mental and behavioural disorders due to psychoactive substance use (F10-19), unemployment approximately four weeks before rehabilitation started, and able to speak German. Premature discontinuation of the regular 12-weeks rehabilitation period was not considered as an exclusion criterion.

### Randomisation procedure

The trial used a quasi-randomised approach of allocating patients to the two study groups. The period of one month was randomly selected for allocating patients recruited in this period to one of the study groups. The group allocation of the first month was implemented by a member of the study coordination centre, who was not involved in participants’ recruitment. In the nine months recruitment period, group allocation alternated monthly at each study site. In two of the four sites, the recruiting team member was blinded towards the alternation procedure. The monthly alternation procedure was used in order to minimise spill-over effects due to informal communication between groups and to keep the initial workload for case managers manageable. Only the case managers in the rehabilitation departments were informed about monthly group allocation and were firmly instructed not to share this information with the therapeutic team.

Six weeks before discharge from inpatient rehabilitation, eligible patients were approached consecutively by a member of the therapeutic team in the rehabilitation department. The number of included patients was limited to 15 per month to keep the workload for case managers manageable. This team member provided a detailed oral and written explanation of the study. Written informed consent was provided on the following workday and baseline assessment was conducted immediately afterwards.

### Intervention: employment-focused case management

The study compared a generalist case management approach focused on return to competitive employment (CMRE - Case Management to improve Return to Employment) to standard care (SC). CMRE was specifically designed to help patients return to work and was added to standard care. It started six weeks before discharge from the rehabilitation department and lasted until 12 months after discharge. CMRE is a manual-based intervention (manual available from the first author), which in our study was carried out by one trained professional in charge in each rehabilitation department, all of whom were experienced in substance use disorder rehabilitation: two qualified social education workers with master degrees, a qualified social worker with a master degree, and an occupational therapist. The focus and amount of CMRE was adjusted to the needs of the individual study participant. After recruitment, the intervention group received an in-depth assessment to identify any assistance needs in work-related and social issues. During inpatient rehabilitation the functions of the case manager were to prepare and to co-ordinate transition from inpatient rehabilitation to competitive employment by collaborating with the multidisciplinary rehabilitation team and local Employment Agencies. After inpatient rehabilitation, CMRE aimed to stabilise the participants’ capability of finding and holding down a job and to engage participants in follow-up services of standard care. Thus, the participant and case manager developed a plan to access follow-up and social services. Monitoring was carried out at least every two weeks at the beginning of the participation, and at least every 4 weeks after stabilization. The case manager documented all activities of the CMRE. In the intervention group no further additional care was offered apart from CMRE and standard care.

The control group received standard care (SC). At the beginning of the study, participants of both groups were already placed in inpatient medical rehabilitation. In Germany, standard treatment of persons with substance use disorders in inpatient medical rehabilitation usually takes between 12 and 15 weeks and an inpatient standardised short-course treatment regimen takes about eight weeks. After inpatient medical rehabilitation, SC in substance use disorders comprised access to a range of services including general practitioners, medical specialist care, low-threshold programs (i.e., first-contact opportunities, street work, supervised drug consumption facilities), addiction advice and treatment centres, detoxification and withdrawal treatment in acute care hospitals, medical inpatient and outpatient rehabilitation, social rehabilitation, self-help groups, and outpatient nursing care. Coordination of these outpatient health services was solely up to the family doctor and the patient or his/her family. In addition, the German pension insurance offers open group meetings to patients after rehabilitation which take place at addiction advice and treatment centres every week or every second week. Twenty sessions lasting 1.5 h are provided over six months. In the case of an imminent crisis one-to-one-sessions are available. Both group meetings and one-to-one-sessions are organised by qualified social workers or psychologists and address themes of preserving abstinence, handling crisis, linking with self-help groups, stabilising social network and use of follow-up services.

### Measures and procedures

Data were collected by use of standardised written interviews at four different assessment time-points: immediately after recruitment, six weeks prior to discharge from inpatient rehabilitation, 1–2 days prior to discharge from inpatient rehabilitation, 12 and 24 months after discharge from rehabilitation. The primary outcome measure was return to work (integration into the primary labour market) at 24 months after discharge from rehabilitation. To assess this parameter, participants were asked whether they had had a paid employment for at least 3 h a day in the last 30 days, with the response options “yes, on the primary labour market”, “yes, a publicly funded employment”, or “no”. Secondary outcomes were abstinence, duration of employment, proportion of publicly funded employment, satisfaction with life, proportion of precarious housing situation, proportion of precarious financial situation, and use of follow-up services. Abstinence was assessed by asking whether there had been substance use since discharge, in the previous 30 days and, when appropriate, how many days with substance use in the last 30 days. Abstinence was classified in ‘abstinence’ (no substance use since discharge from rehabilitation) and ‘abstinence after relapse’ (relapse after discharge but no substance use in the last 30 days). The assessment of satisfaction with life comprised 13 items with a score ranging from 1 “very satisfied” to 6 “very unsatisfied”. It is derived from the German Core Data Set on the Documentation of Addiction Treatment (Client, Catamnesis). Most addiction aid facilities in Germany regularly use the Core Data Set for documentation and evaluation. However the assessment of satisfaction with life is not validated so far. All other secondary outcomes except duration of employment were collected by using the data form of the German Core Data Set on the Documentation of Addiction Treatment (Client). This data set contains items of the “Treatment Demand Indicator“(TDI) of the European Monitoring Centre for Drugs and Drug Addiction (EMCDDA) [11]. In order to reduce response burdens, we restricted data assessment to the Core Data Set questionnaire in combination with a few additional items (i.e., state of employment in the last 30 days and adapted items of employment biography from the German Socio-Economic Panel), and did not apply further instruments in addition to the Core Data Set questionnaire. The Core Data Set and collection procedures are described in detail elsewhere [12].

### Statistical issues

#### Sample size

In the planning phase of the study, register data from the participating departments indicated that in Central Germany approximately 10 % of the unemployed persons discharged from inpatient rehabilitation returned to competitive employment after a one-year period. As an effect of the CMRE-intervention, a 25 % return to work on the primary labour market was expected after 24 months. Therefore, a difference of 15 percentage points between the primary endpoints of the groups regarding was assumed to be clinically relevant for calculating the needed sample size. This margin was suggested by a recent study [10] reporting that after inpatient rehabilitation there had been an increase of 44 % in return to work (including the primary as well as the secondary labour market) in the intervention group compared to the control group. Using a type I error of α = 5 %, a power of 80 %, and a two-sided t-test a sample size of 113 patients per study group was calculated. Since the expected participant dropout rate was estimated to be 30 %, the size of each group was increased by 47 patients, resulting in a total sample size of *n* = 320 (*n* = 160 per study group, *n* = 80 per study site).

#### Methodological approach for analysis

Binary variables are reported using absolute and relative frequencies. For descriptive purposes, continuous variables are reported as means with standard deviations or as medians (10^th^ and 90^th^ percentile) in the case of skewed distributions. Comparisons between groups at baseline were performed using a two-sided t-test.

The analysis of the primary outcome measure at the 12 and the 24-months follow-up was calculated using a linear regression model including the covariates intervention, study site, age, level of education and length of unemployment (at the beginning of the inpatient rehabilitation). All estimates are provided with their 95 % confidence intervals. In addition, for the primary outcome and the outcome abstinence, an intention-to-treat approach was performed using a calculation method from the German Society for Addiction Research and Treatment (Deutsche Gesellschaft für Suchtforschung und Suchttherapie, DGSS). For this analysis, all non-responders at the 12 and 24-months follow-ups were assumed “unemployed” for the primary outcome and “relapsed” for the outcome abstinence.

## Results

### Sample, and follow-up rates

**Fig. 1:**
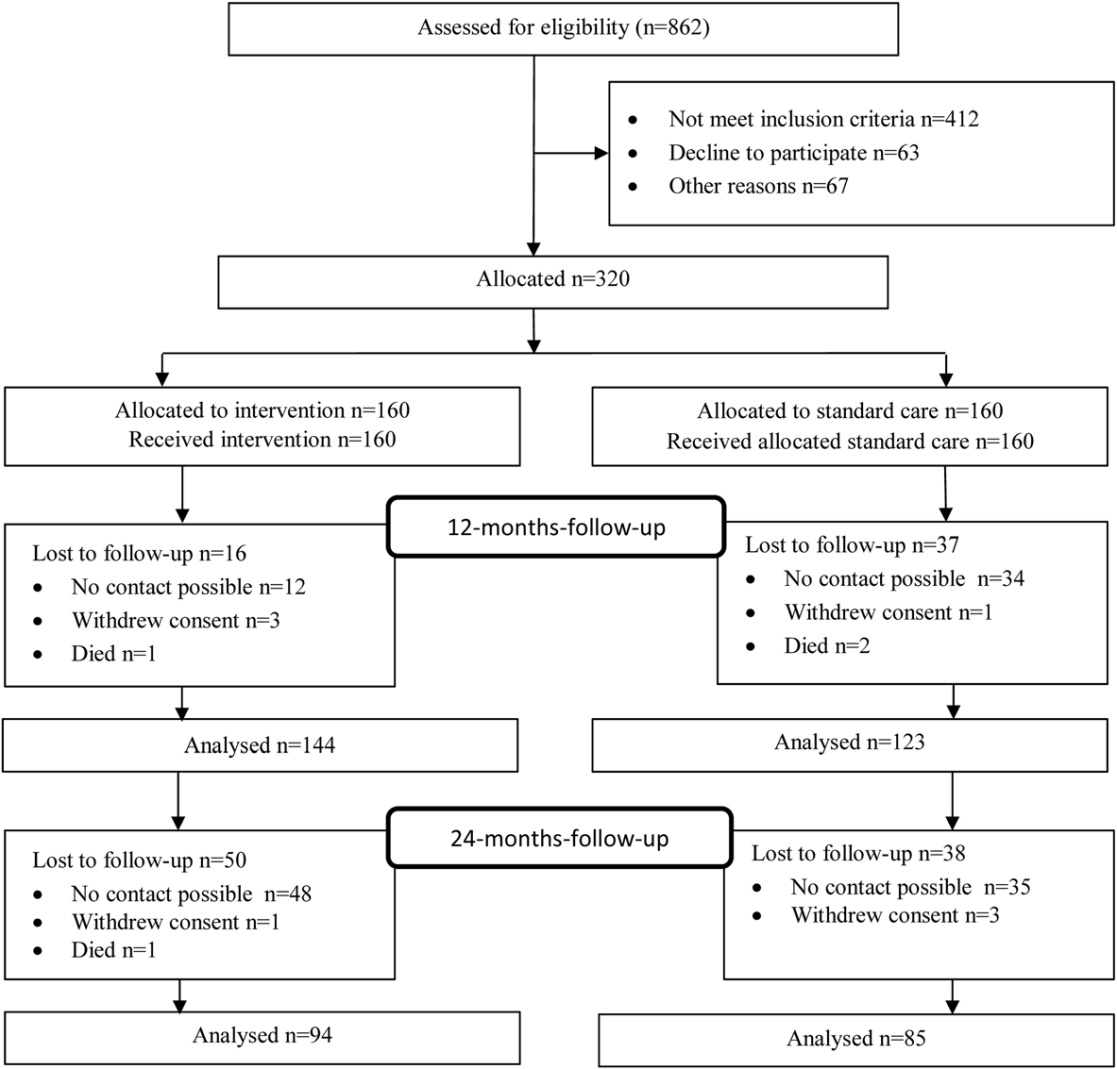
Recruitment and flow through the stages of the trial

Figure 1 displays the flow of the participants through the study. Of the 450 persons who were eligible for the study, 63 declined to participate. A total of 62 eligible patients were not included because the number of 15 patients to be included per month had already been reached. Another reason for not recruiting eligible persons was discharge before recruitment (*n* = 5).

A total of 160 patients were allocated to the intervention group and 160 patients to the control group. The baseline characteristics of the participants are presented in Table 1. There were no statistically significant differences in baseline characteristics between groups.

**Table 1:** Comparison of baseline characteristics

	Intervention *n* = 160		Control *n* = 160		*p*-value
Age, years, mean (SD)	40.96	(10.0)	41.53	(10.1)	0.618
Male gender, *n* (%)	127	(79.4)	139	(86.9)	0.073
Lives alone *n* (%)	95	(59.4)	90	(56.3)	0.571
Lives in a solid relationship *n* (%)	54	(33.8)	57	(35.6)	0.725
Education, highest level, *n* (%)					
Vocational school	119	(74.4)	121	(75.6)	0.796
University (of applied science)	5	(3.1)	6	(3.8)	0.759
Other education level	1	(0.6)	0	(0.0)	0.317
No professional qualification	35	(21.9)	33	(20.6)	0.785
Unemployment at admission, *n* (%)					
Unemployment for less than one year	40	(25.0)	33	(20.6)	0.351
Unemployment for at least one year	116	(72.5)	124	(77.5)	0.302
Others (e.g., imprisonment)	4	(2.5)	3	(1.9)	0.702
Duration of unemployment at admission, weeks mean (SD)	246.86	(303.89)	227.90	(274.18)	0.559
Main diagnosis (ICD-10) *n* (%)					
Alcohol use disorders (F 10)	134	(83.8)	144	(90.0)	0.098
Drug use disorders (F11-F18)	10	(6.3)	4	(2.5)	0.101
Multiple substance use disorders (F19)	16	(10.0)	12	(7.5)	0.429
Number of psychological co-morbidities, m (SD)	1.52	(1.20)	1.50	(1.15)	0.849
Number of psychological and neurological co-morbidities, m (SD)	1.89	(1.32)	1.79	(1.14)	0.442

After 12 months, 90.0 % of the recruited patients still participated in the intervention group compared to 76.9 % in the control group, and after 24 months the follow-up rate was 58.8 % in the intervention group and 53.1 % in the control group. Compared to the group still participating in the study, patients lost at follow-up showed no statistically significant differences regarding age, gender or duration of unemployment at admission.

### Performance of the CMRE

The CMRE was performed for 52.1 (15.0) weeks (mean; SD). The number of contacts between the case manager and the participant was 16 (7; 34) (median; 10^th^ and 90^th^ percentile). The mean contact time (face-to-face or via telephone) over the whole intervention period was 570.9 (473) minutes (mean; SD) per participant. A total of 41.7 % of contacts were face-to face (23.9 % during inpatient rehabilitation, 17.8 % after discharge) and 46.1 % via telephone, the remaining were written communications per email and mail. In addition, case managers realised 7 (2; 21) (median; 10th and 90th percentile) contacts per participant with others than the participant (e.g., service provider, next-of-kin). The most common contact persons were staff members from the regional Employment Agencies with 36.9 % of all contacts, staff members from contact and counselling centres for people with substance abuse (10.6 %), next-of-kin (7.6 %), staff members from institutions providing sheltered housing services (5.3 %), and legal care persons (3.7 %).

### Effectiveness of the CMRE

#### Primary outcome

At the 12-months follow-up 27.7 % of the CMRE group held a job on the primary labour market compared to 30.0 % in the SC group (adjusted Relative Risk 0.90; 95 % Confidence Interval 0.49 to 1.63) (Table 2). At 24-months follow-up, this rate increased to 35.6 % in the CMRE group, and to 41.2 % in the SC group (adjusted Relative Risk 0.92; 95 % Confidence Interval 0.47 to 1.79). Taking non-responders (who were assumed as “unemployed” for the primary outcome) into consideration at the 24-months follow-up, 20.0 % of the CMRE group held a job on the primary labour market compared to 21.9 % in the SC group (adjusted Relative Risk 1.11; 95 % Confidence Interval 0.63 to 1.94).

**Table 2:** Impact of intervention on employment

	12 months after discharge						24 months after discharge					
	Intervention		Control				Intervention		Control			
	*n*	%	*n*	%	RR^a^	(95 % CI)	*n* ^b^	%	*n*	%	RR^a^	(95 % CI)
Competitive employment	38	27.7	36	30.0	0.90	(0.49;1.63)	32	35.6	35	41.2	0.92	(0.47;1.79)
Publicly funded employment	25	18.2	26	21.7	0.81	(0.42;1.57)	11	12.2	10	11.8	0.94	(0.36;2.45)
Unemployment	73	53.7	58	48.3	1.16	(0.70;1.94)	47	52.2	40	47.1	1.13	(0.60;2.13)

^a^adjusted for centre, age, level of education and length of unemployment (at the beginning of the rehabilitation)

^b^participants retired (*n* = 2) and imprisoned participants (*n* = 2) are not included

An additional logistic regression assessed the statistical correlation of the amount of case management time per participant and return to work in the intervention group. The results do not suggest dose-response effect.

#### Secondary outcomes

There were no significant differences between the study groups concerning abstinence, satisfaction with life, housing situation, or precarious financial situation (Table 3). There was also no difference between the study groups concerning the duration of employment. For those who had a job on the primary labour market, the mean number of months in employment in the CMRE group was 6.7 (3.6) (mean; SD) for the first year after discharge and 9.4 (3.5) (mean; SD) for the second year after discharge, compared to the SC group with 7.2 (3.2) and 9.2 (3.8) (mean; SD) months, respectively.

**Table 3:** Abstinence, life satisfaction, precarious housing situation and precarious financial situation 12 and 24 months after discharge

	12 months after discharge					24 months after discharge				
	Intervention		Control		*p* value	Intervention		Control		*p* value
Abstinence *n* (%)	63	(45.7)	71	(57.3)	0.116	47	(50.0)	46	(54.1)	0.735
Abstinence after relapse *n* (%)	33	(23.9)	26	(21.0)	0.663	17	(18.1)	7	(8.2)	0.062
Satisfaction with life mean (SD)										
Sexual partner/spouse	4.2	(2.4)	3.8	(2.4)	0.196	3.2	(2.1)	2.6	(1.6)	0.049
Parents	2.3	(1.2)	2.2	(1.2)	0.535	2.2	(1.4)	2.3	(1.5)	0.706
Children	2.1	(1.4)	2.6	(1.4)	0.032	2.0	(1.3)	2.2	(1.2)	0.516
Friends	2.5	(1.3)	2.4	(1.1)	0.569	2.4	(1.3)	2.4	(1.3)	0.805
Spending free time	2.5	(1.1)	2.5	(1.1)	0.872	2.4	(1.2)	2.4	(1.1)	0.987
Employment	3.3	(1.7)	3.1	(1.7)	0.399	3.2	(1.8)	3.3	(1.9)	0.623
Health	2.6	(1.5)	2.7	(1.5)	0.735	2.7	(1.4)	2.9	(1.4)	0.515
Mental health	2.6	(1.3)	2.6	(1.2)	0.896	2.7	(1.4)	2.6	(1.3)	0.787
Finance	3.3	(1.5)	3.2	(1.4)	0.583	3.2	(1.6)	3.2	(1.4)	0.823
Housing	2.1	(1.2)	2.2	(1.1)	0.771	2.1	(1.0)	2.0	(0.9)	0.707
Offences	1.7	(1.1)	2.0	(1.5)	0.610	1.4	(1.0)	1.2	(0.4)	0.197
Substance use	2.5	(1.5)	2.1	(1.4)	0.149	2.3	(1.3)	2.8	(1.7)	0.242
Daily routine	2.3	(1.2)	2.3	(1.1)	0.958	2.3	(1.3)	2.2	(1.1)	0.641
Housing situation *n* (%)										
In own home	101	(84.2)	88	(88.9)	0.737	62	(89.9)	57	(96.6)	0.148
In supported housing arrangements	7	(5.8)	4	(4.0)	0.481	5	(7.2)	2	(3.4)	0.320
Precarious financial situation *n* (%)	52	(44.1)	41	(44.6)	0.938	21	(30.9)	13	(21.7)	0.242

There was a significantly higher proportion in the CMRE group who immediately after discharge linked with services of the Federal Employment Agency or Job Centres (61.3 %) when compared to the SC group (38.8 %; *p* < 0.001) . There were no significant differences between groups in linkage with other services immediately after discharge, and in use of follow-up services at the 24-months follow-up (please see Additional file 1: Figure S1 and Additional file 2: Figure S2).

## Discussion

This study found that the CMRE was not superior compared to standard care (SC) in its effect on return to work rates of patients with substance use disorders within a 2-years-period after inpatient rehabilitation. Further, CMRE did not show superior effects on abstinence, satisfaction with life, precarious housing situation, precarious financial situation, and duration of employment. There was a significantly higher proportion in the CMRE group, however, which immediately after discharge linked with services of the Federal Employment Agency or Job Centres when compared to the SC group. There were, however, no significant differences between groups in linkage with other services immediately after discharge, and in use of follow-up services at the 24-months follow-up. Thus, our results did not confirm the hypothesis that a CMRE approach might improve the return to work of persons with substance use disorders and could diminish their risk of drug use relapse.

Evaluating CMRE in a multi-site quasi-randomised trial presents several challenges, and this trial had its particular strengths and weaknesses. Due to successful recruitment, implementation of a randomisation procedure resulting in no group differences at baseline assessment, and follow-up rates comparing favourably to those in similar studies in Germany [3, 13], the trial significantly increased the existing evidence base especially in the field of employment-focused outcomes of substance use rehabilitation [6]. Nevertheless, it should be noted, that follow-up rates at 24 months limit the explanatory power of our results. The trial made use of a methodological level up to the one more and more common in mental health services research [14], and increased this level, especially when compared to the pilot study in which the intervention was modelled and tested [10]. Further, the trial used standardised assessment instruments for most outcome domains, demonstrated the feasibility of implementing a manual-based case management intervention providing a close linking between inpatient substance rehabilitation and post-treatment Employment Agencies, and showed that CMRE had an effect on such linkage. Thus, our findings of improved co-operation between rehabilitation services and Employment Agencies confirmed results reported by a previous study [15] on improved linkage with substance abuse treatment as a consequence of case management work. At the post-intervention assessment after 12-months, the drop-out rate in the control group was higher than in the intervention group. These results indicate a potential effect of the CMRE on retention in the study program. This corresponds with findings of a meta-analysis indicating moderate improvements in utilization of substance abuse treatment and important auxiliary services, including retention in substance abuse and auxiliary services [6]. At the 24 months follow-up however, differences in follow-up rates between groups diminished. This suggests an only temporary effect of CMRE on retention in the study program.

The CM approach used in our study might be classified as generalist CM, which is the most frequent approach assessed in trials on patients with substance use disorders [5, 6]. In contrast to factors of success described in the literature when implementing such interventions [16], our approach was not provided by a CM team in each of the participating departments, and did not include the provision of direct services. This could be seen as a potential to optimise our approach when modifying CMRE in the future. Further issues to be improved might be to reduce the high caseload of the case managers in our study, and to increase the rate of face-to-face-contacts above the level achieved in our study, although our approach already resulted in a high rate and time of contacts per participant.

Apart from such practical issues of CMRE provision, we could speculate on some other factors explaining our results that CMRE had no effect on return-to-work rates within a 2-years-follow-up period. Firstly, we would like to point out that our findings are in line with results reported in a most recent meta-analysis on the efficacy of case management, which reported only weak effects on social inclusion [6]. Secondly, contextual factors like the recently significantly decreased unemployment rate in Eastern Germany (from 19.2 % in 2006 to 12.6 % in 2011) [17] might diminish potential effects of the CMRE. Further, a nationwide analysis of catamnestic data of rehabilitation departments (*n* = 5929, provided by the Fachverband Sucht e.V., unpublished data) revealed a lower rate of patients with substance use disorders re-integrated in the primary labour market (30.3 %) than in our study population (48.3 %). This might be due to the already optimised SC in Central Germany. This procedure refers to already established special contracts with Employment Agencies aiming to re-integrate patients from substance use rehabilitation into competitive employment, and therefore might have also decreased the potential effects of the CMRE. The impact of such factors is well established in studies identifying predictors of employment [8], and assessing vocational re-integration after medical rehabilitation of patients [18]. Thirdly, there is no direct influence of the CMRE on the primary outcome of our trial; this influence is mediated via the improved linkage of the patients to the Employment Agencies. Fourthly, in the light of the well-known association of re-integration to competitive work and the decrease of substance consumption and relapse rates, effects of CMRE on abstinence should not be expected if vocational re-integration is not improved. Further, we would like to emphasise that abstinence rates might have been overestimated in our study because of the methodological limitation that patients’ self-reports were used as data sources to assess this issue. Although studies showed a high congruence between self-reports and drug detection tests in urine [19, 20], we cannot exclude the option that our results are biased in this respect by socially desired response behaviour. Fifthly, we can only speculate that results established in the post-interventional period of our study are biased by the higher drop-out rate in the SC group compared to the CMRE group.

## Conclusions

Implications of our trial for further research would be to improve study designs in this field up to the more robust methodological level of simple randomisation, to optimise practical aspects of CMRE provision, and to develop a more profound understanding of factors potentially mediating the effects of CMRE. In our opinion, the MRC framework [21] might be an adequate tool for re-designing CMRE with the aim to focus more clearly on patients’ needs in achieving long-term re-integration into the primary labour market after substance use rehabilitation.

## Abbreviations

CI, confidence interval; CM, case management; CMRE, employment-focused case management; EMCDDA, european monitoring centre for drugs and drug addiction; MRC, medical research council; NCM, non-case-management groups; SC, standard care; SD, standard deviation; TDI, treatment demand indicator

## Additional files

Additional file 1: Figure S1. Linkage with follow-up services immediately after discharge. (JPG 2784 kb) Additional file 2: Figure S2. Use of medical and follow-up services 24 months after discharge. (JPG 2480 kb)
